# Whole plant cannabis extracts in the treatment of spasticity in multiple sclerosis: a systematic review

**DOI:** 10.1186/1471-2377-9-59

**Published:** 2009-12-04

**Authors:** Shaheen E Lakhan, Marie Rowland

**Affiliations:** 1 Global Neuroscience Initiative Foundation, Los Angeles, CA, USA

## Abstract

**Background:**

Cannabis therapy has been considered an effective treatment for spasticity, although clinical reports of symptom reduction in multiple sclerosis (MS) describe mixed outcomes. Recently introduced therapies of combined Δ^9^-tetrahydrocannabinol (THC) and cannabidiol (CBD) extracts have potential for symptom relief with the possibility of reducing intoxication and other side effects. Although several past reviews have suggested that cannabinoid therapy provides a therapeutic benefit for symptoms of MS, none have presented a methodical investigation of newer cannabinoid treatments in MS-related spasticity. The purpose of the present review was to systematically evaluate the effectiveness of combined THC and CBD extracts on MS-related spasticity in order to increase understanding of the treatment's potential effectiveness, safety and limitations.

**Methods:**

We reviewed MEDLINE/PubMed, Ovid, and CENTRAL electronic databases for relevant studies using randomized controlled trials. Studies were included only if a combination of THC and CBD extracts was used, and if pre- and post-treatment assessments of spasticity were reported.

**Results:**

Six studies were systematically reviewed for treatment dosage and duration, objective and subjective measures of spasticity, and reports of adverse events. Although there was variation in the outcome measures reported in these studies, a trend of reduced spasticity in treated patients was noted. Adverse events were reported in each study, however combined TCH and CBD extracts were generally considered to be well-tolerated.

**Conclusion:**

We found evidence that combined THC and CBD extracts may provide therapeutic benefit for MS spasticity symptoms. Although some objective measures of spasticity noted improvement trends, there were no changes found to be significant in post-treatment assessments. However, subjective assessment of symptom relief did often show significant improvement post-treatment. Differences in assessment measures, reports of adverse events, and dosage levels are discussed.

## Background

Spasticity, an involuntary increase in muscle tone or rapid muscle contractions, is one of the more common and distressing symptoms of multiple sclerosis (MS). Medicinal treatment may reduce spasticity, but may also be ineffective, difficult to obtain, or associated with intolerable side effects [1,2]. Cannabis, a psychotropic drug known for its analgesic properties, also has a long history as an effective and tolerable treatment for spasticity [3,4]. Demographic evidence has shown that many people with MS use cannabis for symptom management [5].

Clinical studies, animal models, and anecdotal reports [6-8] have suggested that cannabis may be an effective treatment of MS spasticity. The antispastic effect of cannabis has been supported through a demonstration of the inhibitory properties in exogenous agonists for cannabis receptors found in the CNS [7]. Early clinical trials reporting the efficacy and safety of cannabis use in MS have focused on the effects of Δ^9^-tetrahydrocannabinol (THC). Although these clinical studies reported a therapeutic benefit for MS symptoms, there were concerns of potential intoxication and other side effects of cannabis-based treatment [9]. Another clinical study using a cannabidiol (CBD) extract documented a reduction in spasticity-related pain but not in spasticity [10].

More recent combination therapies using whole plant extracts of both THC and CBD have been introduced and there is evidence that CBD, which is not psychotropic, may reduce THC levels in the brain and attenuate its psychotropic side effects [11-14]. Such therapies may potentially provide a tolerable yet effective treatment for MS symptoms [3]. A number of recent studies [15-22] have investigated the potential efficacy and safety of whole plant extracts of THC and CBD. One of the first large-scale studies of cannabis treatment for MS-related spasticity compared whole plant cannabis extracts with THC and a placebo, and found mixed evidence for the therapeutic benefit of spasticity in MS. A recent review [23] that included a number of these recent studies provided additional support for the benefit of cannabinoids in MS-related spasticity but called for further study into long-term treatment and side effects. A systematic evaluation of recent research had not previously been conducted, and was needed in order to provide organized evidence of cannabinoid treatments and direction for future clinical studies. We therefore systematically reviewed studies that used a combination extract of THC and CBD for the treatment of spasticity.

## Methods

### Searching

We conducted a comprehensive search using MEDLINE/PubMed, Ovid, and CENTRAL (Cochrane Central Register of Controlled Trials) for English-language only literature published from 1999 to April 2009 using different combinations of the following MeSH and free text terms: cannabis, cannabinoid, THC, CBD, multiple sclerosis, spasticity, spasms. Reference lists from retrieved reports were reviewed for additional studies. Unpublished data were not sought and abstracts, letters, case reports, and review articles were excluded. (See Additional file 1 for a Quality of Reporting of Meta-analyses (QUOROM) statement checklist.)

### Selection and quality assessment

Only randomized, placebo-controlled, human studies of shorter treatment periods (under 6 months) were included. Studies were evaluated for methodological quality using Jadad scores [24] and only studies with Jadad scores of 4 or higher were considered for inclusion. Relevant trials included those that had administered a combination THC and CBD extract, those in which clinically stable spasticity had been established prior to trial and those that reported objective measures of pre- and post-treatment spasticity. Studies that used active control groups were not excluded. Abstracts were reviewed for relevancy and full text versions of potentially relevant randomized controlled studies were reviewed. Reports not considered relevant were excluded and all included reports were read in entirety.

### Data abstraction

Data were extracted independently by the authors and any disagreements were resolved by consensus. The following information was extracted from each report: study type, study objective, sample size, controls, type and amount of cannabinoid used, treatment duration, objective and subjective outcome measures, and reported adverse events.

### Analysis

A qualitative summary of the data was completed to compare the various outcome measures used across the included studies. In addition, a quantitative analysis of the one common outcome measure (Ashworth scale) used by the included studies was performed in order to assess statistical heterogeneity.

## Results

### Flow of included studies

**Figure 1:**
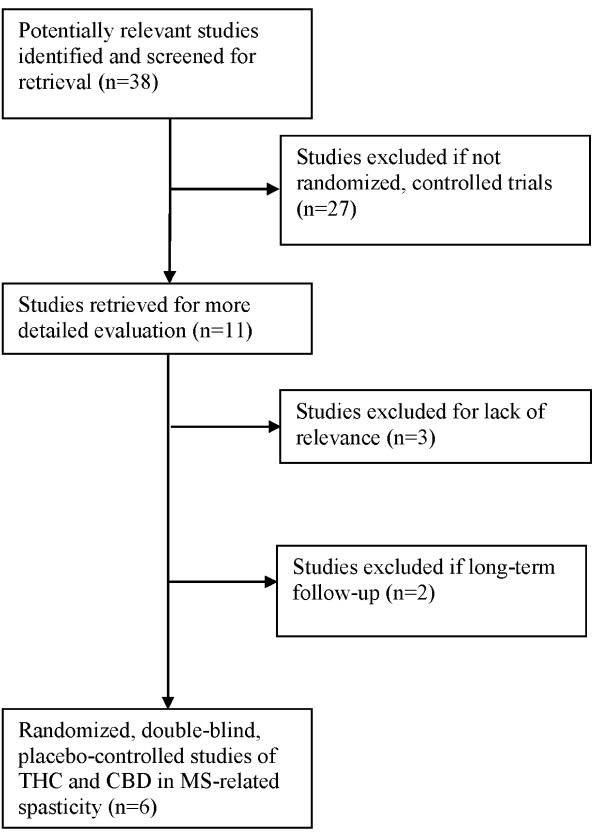
**Flow diagram of included studies**.

Electronic searches found 38 studies that were potentially relevant to the present review. Of these, 33 did not meet the inclusion/exclusion criteria, including 27 reports that were not randomized, controlled trials. One study was excluded for focusing on spasticity-related pain and two were excluded for not assessing the effects of a combined extract of THC and CBD. Two studies were excluded for reporting long-term follow-up data (see Figure 1).

### Study characteristics

Six double-blind, randomized, placebo-controlled trials published between 2002 and 2007 were analyzed [15-20]. These studies included a total of 481 patients with MS who were administered a combined extract of both THC and CBD. Three trials used a crossover design. Three trials used a parallel design in which 339 patients were administered a placebo only. Trial periods ranged from 2 to 15 weeks. Objective spasticity measures were extracted when included in the assessment data from at least two studies. All six trials reported an adjusted mean change score in the Ashworth scale assessment. Other measures of spasticity included mean changes from a baseline score in the following assessments: Visual Analogue Scale (VAS), a rating scale to measure the severity of spasticity; walk time; Rivermead Mobility Index (RMI), a measure of disability related to mobility; and self-reported ratings of spasm frequency or severity. (See Table 1 for the specific characteristics of each reviewed study.)

**Table 1:** Analysis of six randomized controlled trials reporting measures of spasticity after THC-CBD treatment

	**Killestein 2002 **[15]	**Wade 2003 **[17]	**Zajicek 2003 **[16]	**Wade 2004 **[19]	**Vaney 2004 **[18]	**Collin 2007 **[20]
**Design**	Crossover	Crossover	Parallel	Parallel	Crossover	Parallel

**Jaded score**	4	4	5	5	5	4

**Study objective**	Small study to compare effects of THC and THC-CBD	Pilot study to explore benefits for neurogenic symptoms	Large study to compare effects of THC and THC-CBD	Benefits over a range of symptoms	Effects on spasm frequency	Effects on spasticity

**Sample size**	16	14	395 (198 placebo)	154 (77 placebo)	57	184 (64 placebo)

**Duration**	4 weeks	4 weeks	15 weeks	6 weeks	2 weeks	6 weeks

**Intervention**	THC-CBD <10 mg daily	THC-CBD 2.5-120 mg daily	THC-CBD <25 mg daily	THC-CBD <120 mg daily	THC-CBD <30 mg daily	THC-CBD >25 mg daily

**Ashworth score**						

*Mean change*	App. -.3	No change	1.24	-0.37	-2.2	-0.64

*P-value*	Not significant	>0.05	0.29	0.22	0.002	0.218

**VAS spasticity**			Not reported		Not reported	Not reported

*Mean change*	No change	Reduced 14.9 points		Reduced 31.2 points		

*P-value*	Not significant	<0.05		0.001		

**Walk time**	Not reported	Not reported			Not reported	Not reported

*Mean change*			Reduced 4%	Reduced 2.78 (s)		

*P-value*			Not reported	0.07		

**RMI**	Not reported			Not reported		Not reported

*Mean change*		Improved 0.2	Improved 0.4		Improved 0.5	

*P-value*		>0.05	0.21		0.005	

**Self-reported rating**						

*Measure*	Global impression VAS	Numerical symptom scale	Category rating scale	Diary entry VAS scale	Spasm frequency scale	Numerical rating scale

*Mean change*	Worsened	Frequency reduced 1.9; severity reduced 2.1	52% treated reported improvement	Frequency score reduced 21.41; severity reduced 21.67	Reduced 0.4	Reduced 1.18

*P-value*	0.02	<0.05	0.01	0.009	<0.001	0.048

**Adverse events**	41 reported, none serious	16 reported	12 serious reported	4% withdrawn	No serious reported	4.8% withdrawn

RMI, Rivermead Mobility Index; VAS, Visual Analogue Scale.

### Qualitative analysis

#### Overall reduction of spasticity

Five studies [16-20] concluded that cannabis extract may decrease spasticity and improve mobility in patients with MS. One study [15] reported no reduction in spasticity. Adverse effects were reported in each study; however side effects from combined extracts of THC and CBD were generally well-tolerated. Two blinded studies comparing combined extracts of THC and CBD to extracts of THC alone found a lower incidence of adverse events in the combined THC and CBD trials [16,17], and one study found a higher incidence of adverse events [15]. In all three comparison studies, there was no distinction in efficacy between THC extracts and combined THC and CBD extracts.

#### Ashworth score

In one study [18], 50 patients were assessed with the Ashworth scale for muscle tone and showed significant improvement during the active treatment trial. The other five studies reported little to no improvement in their versions of the Ashworth scale. It should be noted that the Ashworth scale is subject to individual assessor evaluation and there may have been variation between studies in the modification of scale measures.

#### Visual Analogue Scale

Three studies reported data from VAS scores [15,17,19]. Two studies reported that patients on active treatment showed a significant improvement in VAS scores [17,19], and one reported no significant difference [15]. In one study [17], daily recorded assessment data from 14 MS patients were mixed with data from six patients with other neurological disorders. The other studies recorded daily [15] or weekly [19] assessments.

#### Walk time

Although five studies included walk time in their proposed assessments, only two studies [16,19] reported data from 160 patients with MS. Although both showed a trend for improvement in walk time, P-value did not reach statistical significance in one study [19] and was not reported in the other [16].

#### Rivermead Mobility Index

Three studies [16-18] reported RMI scores for 275 MS patients. Although there was a trend for improvement from baseline to study completion, mean changes in assessment were significant in one study [18] and insignificant in the other two studies.

#### Other subjective rating scales

All six studies reported an additional measure of subjective assessment. Rating scales were completed by a total of 379 patients in order to record various changes in spasticity throughout the trials. Five studies [16-20] reported significant improvements in spasticity as subjectively rated by patients with MS and one reported deterioration [15].

### Meta-analysis

Three of the studies [15,19,20] did not report adequate (mean and standard deviation) Ashworth scale data for inclusion in the quantitative meta-analysis. This left three studies for the calculation of the pooled mean difference in Ashworth scores. The chi-square test for heterogeneity showed evidence of significant variation between the three studies (χ^2 ^= 5.25, P = .07, l^2 ^= 62%). Given that only three of the six studies reported adequate Ashworth scale data [16-18], of these three only one demonstrated statistically significant findings [18], and the high level of heterogeneity, a quantitative analysis of the data was deemed inappropriate.

## Discussion

### Limitations

There were some limitations to the systematic review. First, this review did not include unpublished data. There may be ongoing clinical trials of combination THC and CBD therapy as it is a relatively recent therapy. There is also the possibility that other clinical reports using whole plant cannabis extracts may have been appropriate for review, but were not included without report of specific methodology. A meta-analytical review of the effects of cannabis on spasticity would be useful, but was not deemed appropriate for the present review because of the variation in assessment data.

### Subjective vs. objective measures

The validity of the Ashworth scale as an outcome measure has been previously questioned [16]. However, we have shown that other objective measures of spasticity (i.e. RMI, walk time) may also fail to adequately support the improvements found in scores from more subjective measures (i.e. rating scales, diary entries). A long-term follow-up study [21] showed a significant improvement in the Ashworth scale, however, the change was still small and was found in the THC group only. Another concern is that participants of both active and placebo trials may not be entirely blind to their treatment status [16], and this may affect subjective assessments. It remains that, without a validated, objective measure of spasticity, it will be difficult to accurately measure the effects of cannabis therapy on MS spasticity.

### Adverse events

Adverse effects were reported in each trial in which patients received active treatment (including THC-only treatment). There is some evidence that combined extracts of THC and CBD may attenuate side effects of THC alone, and future studies are needed to compare the safety of combined cannabis extracts with traditional treatment. Dosage is another concern that should be considered in the context of side effects. Incidence of side effects varies greatly depending on the amount of cannabis needed to effectively limit spasticity. In one study [17], it was noted that the initially permitted dosage level sometimes resulted in marked side effects, and the dosage was thereafter reduced. The careful monitoring of symptom relief and side effects is critical in reaching an individual's optimal dose. Finally, it should be noted that several adverse events were also reported in each trial in which patients received a placebo. In a long-term follow-up [22] of one of the reviewed studies [19], it was determined that most of the reported adverse events were unrelated to cannabis treatment. Considering the distress and limitations spasticity brings to individuals with MS, it would be important to carefully weigh the potential for side effects with the potential for symptom relief, especially in view of the relief reported in subjective assessment.

## Conclusion

We found evidence that combined extracts of THC and CBD may reduce symptoms of spasticity in patients with MS. Although the subjective experience of symptom reduction was generally found to be significant, objective measures of spasticity failed to provide significant changes. In a previous study of spasticity-related pain, MS patients also reported a subjective perception of symptom reduction with cannabinoids [10]. However, since at least one past animal study has provided objective, physiological evidence for the antispastic properties of cannabinoids [7], the distinction between perceived symptom relief and objective physiological changes in humans should therefore be primary in future research efforts.

Given that adverse events occurred in each reviewed trial, we also encourage future comparison studies of cannabis treatments at a wide range of dosage in order to balance potential side effects with maximum therapeutic benefit.

Finally, there is evidence that cannabinoids may provide neuroprotective and anti-inflammatory benefits in MS. Neuroinflammation, found in autoimmune diseases such as MS, has been shown to be reduced by cannabinoids through the regulation of cytokine levels in microglial cells [25]. The therapeutic potential of cannabinoids in MS is therefore comprehensive and should be given considerable attention.

## Abbreviations

CBD: cannabidiol; CENTRAL: Cochrane Central Register of Controlled Trials; MS: multiple sclerosis; QUOROM: quality of reporting of meta-analyses; RMI: Rivermead Mobility Index; THC: Δ^9^-tetrahydrocannabinol; VAS: Visual Analogue Scale.

## Competing interests

The authors declare that they have no competing interests.

## Authors' contributions

SEL and MR participated in the preparation of the manuscript. All authors read and approved the final manuscript.

## Pre-publication history

The pre-publication history for this paper can be accessed here:

http://www.biomedcentral.com/1471-2377/9/59/prepub

## Supplementary Material

Additional file 1 **QUOROM statement checklist**. Quality of Reporting of Meta-analyses (QUOROM) statement checklist. Click here for file
