# Supplementary material for: Whole plant cannabis extracts in the treatment of spasticity in multiple sclerosis: a systematic review
Source: BMC Neurol. 2009 Dec 4;9:59. doi: 10.1186/1471-2377-9-59 (PMC2793241; doi:10.1186/1471-2377-9-59)
Supplement: Additional file 1 — QUOROM statement checklist. Quality of Reporting of Meta-analyses (QUOROM) statement checklist. [file 1471-2377-9-59-S1.PDF]

QUOROM Statement checklist
Whole plant cannabis extracts in the treatment of spasticity in multiple sclerosis: a systematic review

Heading	Subheading	Descriptor	Reported? (Y/N)	Heading: Subheading
Title		Identify the report as a systematic review	Y	Title
Abstract		Use a structured format	Y	Abstract
	Objectives	The clinical question explicitly	Y	Abstract: Background
	Data sources	The databases (ie, list) and other information sources	Y	Abstract: Methods
	Review methods	The selection criteria (ie, population, intervention, outcome, and study design); methods for validity assessment, data abstraction, and study characteristics, and quantitative data synthesis in sufficient detail to permit replication	Y	Abstract: Methods
	Results	Characteristics of the RCTs included and excluded; qualitative and quantitative findings (ie, point estimates and confidence intervals); and subgroup analyses	Y	Abstract: Results
	Conclusion	The main results	Y	Abstract: Conclusion
		Describe		
Introduction		The explicit clinical problem, biological rationale for the intervention, and rationale for review	Y	Introduction
Methods	Searching	The information sources, in detail (eg, databases, registers, personal files, expert informants, agencies, hand-searching), and any restrictions (years considered, publication status, language of publication)	Y	Methods: Searching
	Selection	The inclusion and exclusion criteria (defining population, intervention, principal outcomes, and study design	Y	Methods: Selection and quality assessment
	Validity assessment	The criteria and process used (eg, masked conditions, quality assessment, and their findings)	Y	Methods: Selection and quality assessment
	Data abstraction	The process or processes used (eg, completed independently, in duplicate)	Y	Methods: Data abstraction
	Study characteristics	The type of study design, participants' characteristics, details of intervention, outcome definitions, and how clinical heterogeneity was assessed	Y	Methods: Data abstraction, Analysis
	Quantitative data synthesis	The principal measures of effect (eg, relative risk), method of combining results (statistical testing and confidence intervals), handling of missing data; how statistical heterogeneity was assessed; a rationale for any a-priori sensitivity and subgroup analyses; and any assessment of publication bias	Y	Methods: Analysis (Note: quantitative data synthesis was deemed inappropriate and therefore not pursued.)
Results	Trial flow	Provide a meta-analysis profile summarising trial flow (see figure)	Y	Results: Flow of included studies, Figure 1
	Study characteristics	Present descriptive data for each trial (eg, age, sample size, intervention, dose, duration, follow-up period)	Y	Results: Study characteristics, Table 1
	Quantative data synthesis	Report agreement on the selection and validity assessment; present simple summary results (for each treatment group in each trial, for each primary outcome); present data needed to calculate effect sizes and confidence intervals in intention-to-treat analyses (eg 2X2 tables of counts, means and SDs, proportions)	Y	See note above under Methods >> Quantitative data synthesis
Discussion		Summarise key findings; discuss clinical inferences based on internal and external validity; interpret the results in light of the totality of available evidence; describe potential biases in the review process (eg, publication bias); and suggest a future research agenda	Y	Discussion
